# Efficient spin filter using multi-terminal quantum dot with spin-orbit interaction

**DOI:** 10.1186/1556-276X-6-436

**Published:** 2011-06-22

**Authors:** Tomohiro Yokoyama, Mikio Eto

**Affiliations:** 1 Faculty of Science and Technology, Keio University, 3-14-1 Hiyoshi, Kohoku-ku, Yokohama 223-8522, Japan

## Abstract

We propose a multi-terminal spin filter using a quantum dot with spin-orbit interaction. First, we formulate the spin Hall effect (SHE) in a quantum dot connected to three leads. We show that the SHE is significantly enhanced by the resonant tunneling if the level spacing in the quantum dot is smaller than the level broadening. We stress that the SHE is tunable by changing the tunnel coupling to the third lead. Next, we perform a numerical simulation for a multi-terminal spin filter using a quantum dot fabricated on semiconductor heterostructures. The spin filter shows an efficiency of more than 50% when the conditions for the enhanced SHE are satisfied.

PACS numbers: 72.25.Dc,71.70.Ej,73.63.Kv,85.75.-d

## Introduction

The injection and manipulation of electron spins in semiconductors are important issues for spin-based electronics, "spintronics."[1] The spin-orbit (SO) interaction can be a key ingredient for both of them. The SO interaction for conduction electrons in direct-gap semiconductors is written as(1)

where *U*(***r***) is an external potential, and ***σ ***indicates the electron spin ***s ***= ***σ***/2. The coupling constant *λ *is largely enhanced in narrow-gap semiconductors such as InAs, compared with the value in the vacuum [2].

In two-dimensional electron gas (2DEG; *xy *plane) in semiconductor heterostructures, an electric field perpendicular to the 2DEG, , induces the Rashba SO interaction [3,4](2)

where . The Rashba SO interaction can be tuned by the external electric field, or the gate voltage [5-7]. In the spin transistor proposed by Datta and Das [8], electron spins are injected into the 2DEG from a ferromagnet, and manipulated by tuning the strength of Rashba SO interaction. However, the spin injection from a ferromagnetic metal to semiconductors is generally not efficient, less than 0.1%, because of the conductivity mismatch [9]. To overcome this difficulty, the SO interaction may be useful for the spin injection into semiconductor without ferromagnets. Several spin filters were proposed utilizing the SO interaction, e.g., three-terminal devices based on the spin Hall effect (SHE) [10-12], a triple-barrier tunnel diode [13], a quantum point contact [14,15], and an open quantum dot [16-19].

The SHE is one of the phenomena utilized to create a spin current in the presence of SO interaction. There are two types of SHE. One is an intrinsic SHE which creates a dissipationless spin current in the perfect crystal [20-22]. The other is an extrinsic SHE caused by the spin-dependent scattering of electrons by impurities [23-25]. In our previous articles [26-28], we have formulated the extrinsic SHE in semiconductor heterostructures with an artificial potential created by antidot, scanning tunnel microscope (STM) tip, etc. The artificial potential is electrically tunable and may be attractive as well as repulsive. We showed that the SHE is significantly enhanced by the resonant scattering when the attractive potential is properly tuned. We proposed a multi-terminal spin filter including the artificial potential, which shows an efficiency of more than 50% [27].

**Figure 1:**
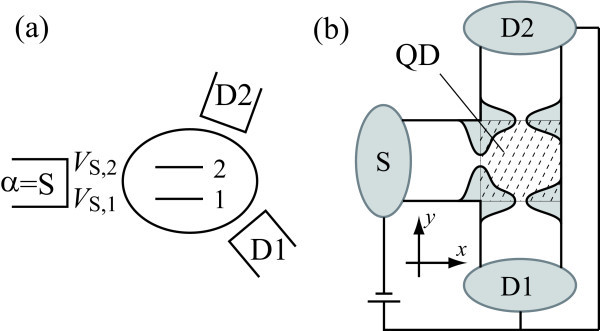
**Models of a multi-terminal spin filter using a quantum dot with SO interaction**. **(a) **Impurity Anderson model with three leads. There are two energy levels (*j *= 1, 2) in the quantum dot. They are connected to lead by tunnel coupling, *V_α,j _***(b) **A three-terminal spin-filtering device fabricated on semiconductor heterostructures. 2DEG is confined in the *xy *plane. A quantum dot is formed by quantum point contacts on three leads. Reservoir S is a source from which spin-unpolarized electrons are injected into the quantum dot. The voltage is identical in reservoirs D1 and D2.

In the present article, we investigate an enhancement of the SHE by the resonant tunneling through a quantum dot (QD) with strong SO interaction, e.g., InAs QD [29-34]. The QD shows a peak structure of the current as a function of gate voltage, the so-called Coulomb oscillation. At the current peaks, the resonant tunneling takes place at low temperatures. First, we consider an impurity Anderson model with three leads, as shown in Figure 1a. There are two energy levels in the QD. We show a remarkable enhancement of the SHE when the level spacing in the QD is smaller than the level broadening. The SHE is electrically tunable by changing the tunnel coupling to the third lead.

Next, we perform a numerical simulation for a spin-filtering device fabricated on semiconductor heterostructures, in which a QD is connected to three leads (Figure 1b). The device is described using the tight-binding model of square lattice, which discretizes the two-dimensional space [35]. We find that the spin filter indicates an efficiency of more than 50% when some conditions are satisfied.

## Formulation of spin Hall effect

To formulate the SHE in a multi-terminal QD, we begin with an impurity Anderson model shown in Figure 1a. The number of leads is denoted by *N *(*N *≥ 2). As a minimal model, we consider two energy levels in the QD; *ε*_1_, and *ε*_2_. We assume that the wavefunctions, *ψ*_1 _and *ψ*_2_, in the QD are real in the absence of a magnetic field. Since the SO interaction (1) includes the momentum ***p ***= -*iħ***∇**, which is a pure imaginary operator, the diagonal elements of the SO interaction, 〈*j*|*H*_SO_|*j*〉 (*j *= 1, 2), disappear. The off-diagonal elements are denoted by

for spin ±1/2 in the direction of 〈2|(***p ***× **∇***U*)|1〉.

The state |*j*〉 in the QD is connected to lead *α *by tunnel coupling, *V*_*α*,*j *_(*j *= 1, 2). The strength of the tunnel coupling is characterized by the level broadening, Γ*_α _*= *πν_α _*(*V*_*α*,1_^2 ^+ *V*_*α*,2_^2^), where *ν_α _*is the density of states in the lead. The leads have a single channel of conduction electrons. Unpolarized electrons are injected into the QD from source lead (*α *= S) and output to drain leads (D*n*; *n *= 1, 2, ⋯, *N *- 1). The electric voltage is identical in the (*N *- 1) drain leads. The current to the drain D*n *of each spin component, *I*_*n*,±_, is generally formulated in terms of Green functions in the QD [36].

We formulate the SHE in the vicinity of the Coulomb peaks where the resonant tunneling takes place. Neglecting the electron-electron interaction, we obtain an analytic expression of the conductance *G*_*n*,± _for spin ±1/2 [37]. We find that the SHE is absent (G_1,+ _= G_1,-_) when the number of leads is *N *= 2, as pointed out by other groups (see Ref. [18] and related references cited therein). For *N *= 3, the conductance to lead D1 is given by(3)

Here, *D *is the determinant of the QD Green function, which is independent of spin ±1/2 (see Ref. [37] for detail). We introduce unit vectors, ***e***_*α*_(*α *= S, D1, and D2), where , in the pseudo-spin space representing levels 1 and 2 in the QD. (***a ***× ***b***)_3 _= *a*_1_*b*_2 _- *a*_2_*b*_1_.

In Equation 3, the spin current [∝ (*G*_*n*,+ _- *G*_*n*,-_)] stems from the interplay between SO interaction, Δ_SO_, and tunnel coupling to lead D2, Γ_D2_. We exclude specific situations in which two from ***e***_S_, ***e***_D1_, and ***e***_D2 _are parallel to each other hereafter. We find the conditions for a large spin current as follows: (i) The level spacing, Δ*ε *= *ε*_2 _- *ε*_1_, is smaller than the level broadening by the tunnel coupling to leads S and D1, Γ_S _+ Γ_D1_. (ii) The Fermi level in the leads is close to the energy levels in the QD (resonant condition). (iii) The level broadening by the tunnel coupling to lead D2, Γ_D2_, is comparable with the strength of SO interaction Δ_SO_.

**Figure 2:**
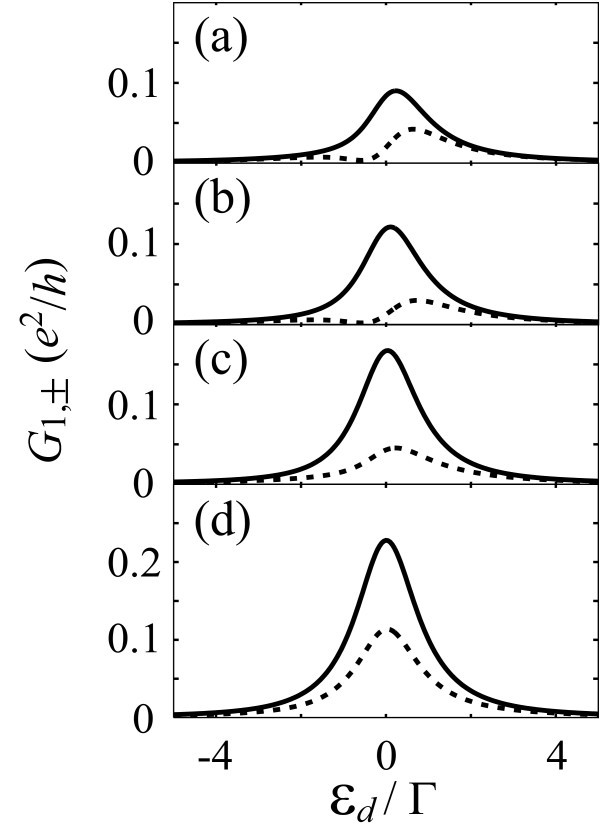
**Calculated results of the conductance ***G*_1,± _**to the drain 1 for spin **±1/2 **in the impurity Anderson model with three leads**. In the abscissa, *ε_d _*= (*ε*_1 _+ *ε*_2_)/2, where *ε*_1 _and *ε*_2 _are the energy levels in the quantum dot. Solid and broken lines indicate *G*_1,+ _and *G*_1,-_, respectively. The level broadening by the tunnel coupling to the source and drain 1 is Γ_S _= Γ_D1 _≡ Γ (*V*_S,1_/*V*_S,2 _= 1/2, *V*_D1,1_/V_D1,2 _= -3), whereas that to drain 2 is (a) Γ_D2 _= 0.2Γ, (b) 0.5Γ, (c) Γ, and (d) 2Γ (*V*_D2,1_/*V*_D2,2 _= 1). Δ*ε *= *ε*_2 _- *ε*_1 _= 0.2Γ. The strength of SO interaction is Δ_SO _= 0.2Γ.

Figure 2 shows the conductance of each spin, *G*_1,+ _(solid line) and *G*_1,- _(broken line), as a function of *ε_d _*= (*ε*_1 _+*ε*_2_)/2, in the case of N = 3. The conductance shows a peak reflecting the resonant tunneling around the Fermi level in the leads, which is set to be zero. We set Γ_S _= Γ_D1 _≡ Γ, whereas (a) Γ_D2 _= 0.2Γ, (b) 0.5Γ, (c) Γ, and (d) 2Γ. The level spacing in the QD is Δ*ε *= 0.2Γ. The strength of SO interaction is Δ_SO _= 0.2Γ. The calculated results clearly indicate that the SHE is enhanced by the resonant tunneling around the peak. We obtain a large spin current when Γ_D2 _~ Δ_SO_, as pointed out previously. Therefore, the SHE is tunable by changing the tunnel coupling to the third lead, Γ_D2_.

## Numerical simulation

To confirm the enhancement of SHE discussed using a simple model, we perform a numerical simulation for a spin-filtering device in which a QD is connected to three leads, as shown in Figure 1b. 2DEG in the *xy *plane is formed in a semiconductor heterostructure. Reservoir S is a source from which spin-unpolarized electrons are injected into the QD. The voltage is identical in reservoirs D1 and D2.

## Model

A QD is connected to reservoirs through quantum wires of width *W*. A hard-wall potential is assumed at the edges of the quantum wires. The QD is formed by quantum point contacts on the wires. The potential in a quantum wire along the *x *direction is given by [38](4)

with(5)

where *θ*(*t*) is a step function [*θ *= 1 for *t *> 0, and *θ *= 0 for *t *< 0], *U*_0 _is the potential height of the saddle point. The parameter Δ characterizes the confinement in the *y *direction, whereas *L *is the thickness of the potential barrier. When the electrostatic energy in the QD is changed by the gate voltage *V*_g_, the potential is modified to *U*(*x*, *y*, *U*_0 _- *eV*_g_)+*eV*_g _inside the QD region [netted square region in Figure 1b] and *U*(*x*, *y*, *U*_0_) outside of the QD region (The potential in the three quantum wires is overlapped by each other inside the QD region. Thus, we cut off the potential at the diagonal lines in the netted square region in Figure 1b).

The gradient of *U *gives rise to the SO interaction in Equation 1, as(6)

Although the SO interaction is also created by the hard-wall potential at the edges of the leads, it is negligible because of a small amplitude of the wavefunction there [27].

The device is described using the tight-binding model of square lattice, which discretizes the real space in two dimensions [35,38]. The width of the leads is *W *= 30*a*, with lattice constant *a*. The effective mass equation including the SO interaction in Equation 6 is solved numerically. The Hamiltonian is(7)

where  and *c*_*i,j*;*σ *_are creation and annihilation operators of an electron, respectively, at site (*i*, *j*) with spin *σ*. *t *= *ħ*^2^/(2m* *a*^2^), and m* is the effective mass of electrons.  is the potential energy at site (*i*, *j*), in units of *t*. The transfer term in the *x *direction is given by(8)

whereas that in the *y *direction by(9)

with .  is the potential energy at the middle point between the sites (*i*, *j*) and (*i *+ 1, *j*), and  is that of (*i*, *j*) and (*i*, *j *+ 1).

We introduce a random potential *w_i,j _*in the QD region. -*W*_ran_/2 ≤ *w_i,j _*≤ *W*_ran_/2. The randomness *W*_ran _is related to the mean free path Λ by the following equation [38]:(10)

We disregard the SO interaction induced by the random potential.

We assume that the Fermi wavelength is *λ*_F _= *W*/3 = 10*a*. The strength of SO interaction is , which corresponds to the value for InAs, *λ *= 117.1 Å^2 ^[2], with the width of the leads *W *= 30*a *≈ 50 nm. The Fermi energy is given by *E*_F_/*t *= 2 - 2 cos(*k*_F_*a*), with *k*_F _= 2*π*/*λ*_F_. The thickness of tunnel barriers is *L*/*λ*_F _= 2. The randomness is *W*_ran_/*E*_F _= 1, which means that the mean free path is Λ/λ_F _≈ 19.4. The temperature is *T *= 0.

## Calculated results

Since the *z *component of spin is conserved with the SO interaction (6), we can evaluate the conductance for *s_z _*= ±1/2 separately. Using the Green's function and Landauer-Büttiker formula, we calculate the conductance  from reservoir *α *to reservoir *β*, for spin *s_z _*= ±1/2 [35,38,39]. The total conductance is , whereas the spin polarization in the *z *direction is given by(11)

We focus on the transport from reservoir S to D1 and omit the superscripts (*β *= D1, *α *= S) of  and *P_z_^βα^*.

**Figure 3:**
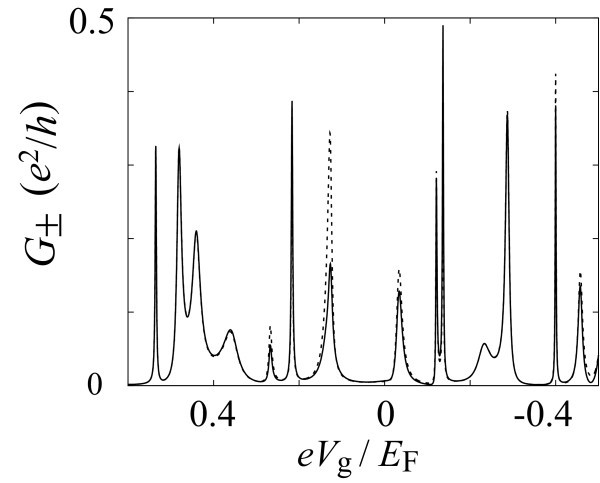
**Results of the numerical simulation for the spin-filtering device shown in Fig. 1(b)**. The conductance *G*_± _for spin *s_z _*= ±1/2 from reservoir S to D1 is shown as a function of gate voltage *V*_g _on the quantum dot. Solid and broken lines indicate *G*_+ _and *G*_-_, respectively. The height of the tunnel barriers is *U*_S _= *U*_D1 _= *U*_D2 _= 0.9*E*_F_.

Figure 3 presents the conductance *G*_± _for spin *s_z _*= ±1/2 as a function of the gate voltage *V*_g _on the QD. We choose *U*_S _= *U*_D1 _= *U*_D2 _= 0.9*E*_F _for the tunnel barriers. The conductance *G*_+ _(solid line) and *G*_- _(broken line) reflect the resonant tunneling through discrete energy levels formed in the QD region. Around some conductance peaks, e.g., at *eV*_g_/*E*_F _≈ 0.13 and -0.03, the difference between *G*_+ _and *G*_- _is remarkably enhanced. Thus, a large spin current is observed, which implies that two energy levels are close to each other around the Fermi level there.

**Figure 4:**
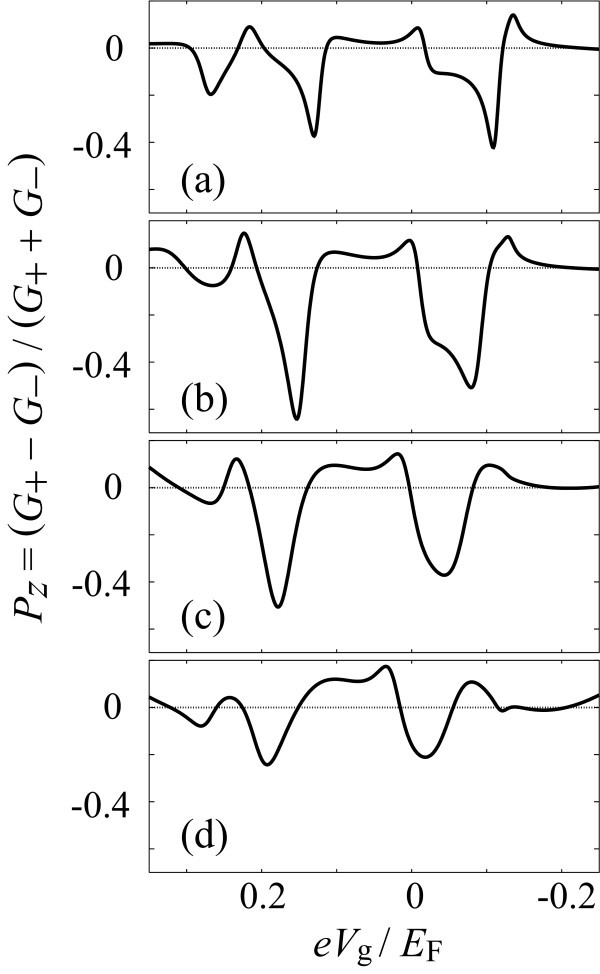
**Results of the numerical simulation for the spin-filtering device shown in Fig. 1b**. The spin polarization *P_z _*of the output current in reservoir D1 is shown as a function of gate voltage *V*_g _on the quantum dot. The height of the tunnel barriers is *U*_S _= *U*_D1 _= 0.9*E*_F_, whereas (a) *U*_D2_/*E*_F _= 0.9, (b) 0.8, (c) 0.7, and (d) 0.6.

The spin polarization *P_z _*is shown in Figure 4a for the range of 0.35 >*eV*_g_/*E*_F _> -0.25. Around the conductance peaks, a large spin polarization is observed. The efficiency of the spin filter becomes 37% at *eV*_g_/*E*_F _≈ 0.13 and 42% at *eV*_g_/*E*_F _≈ -0.03.

Next, we examine the tuning of the spin filter by changing the tunnel coupling to lead D2. In Figure 4, we set (b) *U*_D2_/*E*_F _= 0.8, (c) 0.7, and (d) 0.6 while both *U*_S _and *U*_D1 _are fixed at 0.9*E*_F_. As *U*_D2 _is decreased, the tunnel coupling becomes stronger. First, the spin polarization increases with an increase in the tunnel coupling. It is as large as 63% in the case of Figure 4b. With an increase in the tunnel coupling further, the spin polarization decreases (Figure 4c,d).

## Conclusions

We have formulated the SHE in a multi-terminal QD. The SHE is enhanced by the resonant tunneling through the QD when the level spacing is smaller than the level broadening. We have shown that the SHE is tunable by changing the tunnel coupling to the third lead. Next, the numerical simulation has been performed for a spin-filtering device using a multiterminal QD fabricated on semiconductor heterostructures. The efficiency of the spin filter can be larger than 50%.

## Abbreviations

QD: quantum dot; STM: scanning tunnel microscope; SHE: spin Hall effect; SO: spin-orbit.

## Competing interests

The authors declare that they have no competing interests.

## Authors' contributions

TY participated the discussion of the analytical model and carried out the numerical calculation. ME carried out the analytical formulation of spin Hall effect. All authors conceived of the study, drafted the manuscript, read and approved the final manuscript.
